# A rapid and efficient method for uniform gene expression using the barley stripe mosaic virus

**DOI:** 10.1186/s13007-017-0175-5

**Published:** 2017-04-11

**Authors:** Arnaud Cheuk, Mario Houde

**Affiliations:** Département des sciences biologiques, Centre TOXEN, Université du Québec à Montréal, Montreal, QC H3C 3P8 Canada

**Keywords:** Barley stripe mosaic virus (BSMV), Gene overexpression, Wheat, Barley, *Brachypodium*, Maize, Arabidopsis, *Nicotiana benthamiana*

## Abstract

**Background:**

The barley stripe mosaic virus (BSMV) has become a popular vector to study gene function in cereals. However, studies have been limited to gene silencing in leaves of barley or wheat. In addition, the method produces high variability between different leaves and plants. To overcome these limitations, we explored the potential of modifying the inoculation protocol for BSMV gene overexpression. An improved light, oxygen or voltage-sensing (iLOV) domain-based fluorescent protein was used as a reporter of gene expression to monitor the infection and spread of BSMV. Tobacco (*Nicotiana benthamiana*) leaves were infected via agroinfiltration and the leaves were homogenized to extract the BSMV particles and inoculate wheat tissues using the traditional leaf abrasion method or by incubation during seed imbibition in a Petri dish.

**Results:**

Compared to the leaf abrasion method, the seed imbibition method resulted in a high and uniform detection of iLOV in both roots and leaves of different wheat cultivars and other monocot and dicot species within 7 days after germination. The progression of viral infection via the imbibition method as measured by the expression of iLOV was more stable in different organs and tissues and is transmissible to the next generation.

**Conclusion:**

Our results show that BSMV can be used as a vector for the expression of small genes such as *iLOV* in wheat roots and leaves. The inoculation by seed imbibition allows genes to be expressed rapidly and uniformly in wheat and different monocot and dicot species compared to the traditional leaf abrasion method. It also produces high successful transformation as early as 7 days post infection allowing gene function studies during the first generation of infected plants. Furthermore, the method is simple, rapid, and inexpensive compared to the production of transgenic plants.

**Electronic supplementary material:**

The online version of this article (doi:10.1186/s13007-017-0175-5) contains supplementary material, which is available to authorized users.

## Background

As a result of rapid advances in genomics, the availability of powerful tools for gene function analysis has become a necessity, especially for important crops. In recent years, barley stripe mosaic virus (BSMV) vectors have been modified and showed important applications for high throughput assays due to its simplicity and ease of use [1]. The BSMV virus is a tripartite (α, β, γ) positive strand RNA virus with RNAs capped at the 5′ end [2]. These RNAs encodes all the genes necessary for viral RNA replication and viral propagation [3]. Recently, a BSMV vector system coupling a ligation independent cloning strategy with an *Agrobacterium tumefaciens*-mediated delivery system has been engineered and provides substantial advantages in expense, cloning efficiency and ability to apply virus-induced gene silencing (VIGS) for high throughput genomic studies [2]. However, the potential of BSMV vector for gene overexpression in wheat and the optimal inoculation protocol has been less studied [1, 4]. Virus-mediated overexpression (VOX) has been demonstrated for the first time in studies involving the green fluorescent protein (GFP) as reporter [1, 5, 6]. While GFP expression from BSMV was shown to be strong, studies reported that its overall level of expression in monocotyledonous plants is patchy [1, 4, 6]. It was suggested that this could be due to the relatively large size of GFP (720 bp), limiting local and systemic spread of BSMV, and causing instability in the viral genome [1]. Insert sizes lesser than 500 bp were shown to be relatively more stable than larger inserts [1]. An alternative strategy to verify the potential of BSMV vector for a more uniform and stable gene overexpression would be to use a smaller fluorescent protein to reduce gene size and increase stability. An improved light, oxygen or voltage-sensing (iLOV) domain of the plant blue light receptor, phototropin fluorescent protein has previously been demonstrated to be more stable compared to GFP in wheat leaves using BSMV [1]. However, several cells containing chloroplasts do not express iLOV, showing that the expression is not uniform. This is probably due to the use of the leaf abrasion method of virus inoculation [1].

Furthermore, most protocols of VIGS involving the use of BSMV in plants are so far established for gene silencing in vegetative tissues with very limited success in infection at an early stage resulting in difficulties to evaluate the impact of gene silencing since results vary from different tissue samples [2, 7–9]. Transmission of BSMV through seeds allows a more uniform gene expression in different tissues without showing any viral symptoms [6]. Based on this observation, we postulated that inoculation with BSMV particles at the earliest time possible may result in a more uniform gene expression. We have thus developed a new procedure of BSMV inoculation at the seed imbibition stage. This modification allows rapid and efficient gene overexpression in different wheat tissues, genotypes, and in different monocot and dicot species.

## Methods

### Plant material and growth conditions

*Nicotiana benthamiana* used for agroinfiltration was grown in a controlled environment chamber at 24 °C under 14 h photoperiod, 100 μmol m^−2^ s^−1^ (fluorescent and incandescent lighting) and 70% relative humidity. Different plant species and cultivars were used for BSMV inoculation: *Triticum aestivum* (wheat) cv. Atlas66, Norstar, *Hordeum vulgare* (barley) cv. Sophie, *Zea mays* (maize) cv. Pioneer Hybrid 3921, *Brachypodium distachyon* inbred line Bd21, *N. benthamiana* (tobacco) and *Arabidopsis thaliana* ecotype Columbia (Col-0).

### Construction of *Agrobacterium*-mediated BSMV-*iLOV* vector

**Fig. 1:**
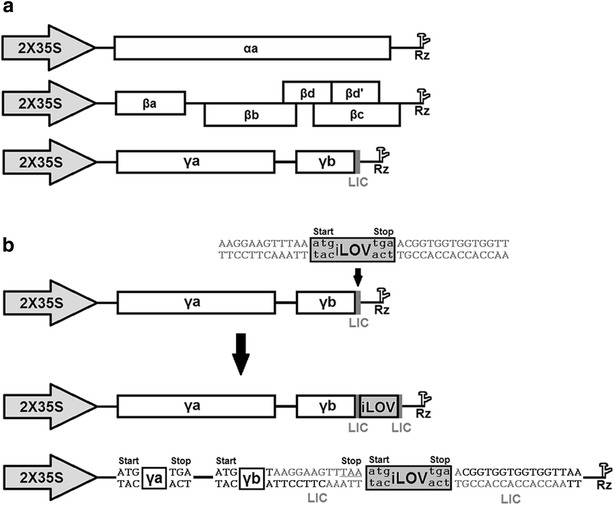
Construction of Agrobacterium-mediated BSMV:*iLOV* vector with the LIC cloning site. **a** Schematic representation of pCaBS-α, pCaBS-β and pCaBS-γ. BSMV α, β and γ cDNAs were inserted between the double 35S promoter and a ribozyme sequence (Rz). **b** Cloning of *iLOV* cDNA (330 bp) in pCaBS-γ via LIC cloning

The three plasmids pCaBS-α, pCaBS-β and pCaBS-γ comprising respectively the tripartite genome (RNAα, RNAβ, and RNAγ) of the BSMV strain ND18 were selected for *iLOV* overexpression (Fig. 1a). The *iLOV* coding sequence (330 bp) was amplified by PCR from pGEX *iLOV* (Addgene, plasmid #26587) using the Q5 high fidelity DNA polymerase (New England Biolabs) and the following primers containing the LIC sites (underlined): AAGGAAGTTTAAATGATAGAGAAGAATTTCGTCATCACT (forward) and CAACCACCACCACCGTCTATACATGATCACTTCCATCGAGCTG (reverse). PCR products were purified and cloned into the pCaBS-γbLIC via ligation independent cloning (LIC) as described by Yuan et al. [2] (Fig. 1b; Additional file 1: Fig. S1). The construct was then introduced into *Escherichia coli* DH10B. Positive colonies were identified by colony PCR, endonuclease digestion, and confirmed by sequencing.

### Agroinfiltration of *N. benthamiana* and viral inoculation

The pCaBS-α, pCaBS-β and pCaBS-γb:*iLOV* were introduced into *A. tumefaciens* strain EHA105 as described previously by Yuan et al. [2]. Equal amounts of *Agrobacterium* harboring pCaBS-α, pCaBS-β and pCaBS-γb:*iLOV* were mixed (ratio 1:1:1) and incubated for 3–5 h at 28 °C. Infiltration of *N. benthamiana* leaves was performed using a 1-ml needleless syringe. The bacterial mixture was infiltrated into 3–6 spots (100 µl of resulting in approximately 3–4 cm^2^ infiltrated area) on each *N. benthamiana* leaf. After agro-infiltration, *N. benthamiana* plants were maintained in a controlled environment chamber. At 7 days post-infiltration (dpi), 0.5 g of spot-agroinfiltrated leaves were harvested and ground in 1 ml of 20 mM Na-phosphate buffer (pH 7.2) using a mortar and pestle. Homogenates can be directly used for virus inoculation or aliquoted in small volumes and stored at −20 °C for later use. Two methods of inoculation were tested in this study. Mechanical inoculation was performed using the traditional leaf abrasion method [2, 10]. Diatomaceous earth (1%, w/v) was added to the homogenate for mechanical inoculation onto 7-days old wheat leaves (first leaf). For the seed imbibition method, seeds of *T. aestivum*, *H. vulgare*, *Z. mays*, *B. distachyon* were germinated in a Petri dish containing *N. benthamiana* homogenate and distilled water. Different ratios of homogenate: distilled water were tested and a ratio of 1:100 (v/v) was used in this study, since this ratio did not lead to any alterations of seed germination and seedling development. Two different eudicot species (*A. thaliana* and *N. benthamiana*) were grown from seeds on 0.8% agarose and the diluted homogenate was directly spread on agarose after 3 days of seed stratification at 4 °C.

### Quantitative RT-PCR analysis

Total RNA (including viral RNA) was isolated from different plant tissues using the RNeasy plant mini kit (Qiagen) and treated with on-column RNase free DNAase (Qiagen) before the reverse transcription step. Real-time quantitative RT-PCR was performed on a CFX96 Touch™ Thermal cycler (Bio-Rad) using the SsoFast EvaGreen Supermix (Bio-Rad). The *iLOV* RNA region was amplified by RT-PCR using the following primers ACAGATCAAGCGACTGTCCA (forward) and CACAGGTTGCAGGTGGAGTA (reverse). Amplification was performed with the following thermal cycling conditions: 5 min at 95 °C followed by 35 cycles of 95 °C for 15 s, 58 °C for 30 s. The copy number of BSMV*:iLOV* in infected tissue was normalized to the 18S *rRNA* and determined from a calibration curve using known amounts of *iLOV* cDNA as described previously [11].

### Fluorescence imaging

iLOV fluorescence images were obtained using a confocal laser-scanning microscope (Nikon inverted microscope Eclipse T*i*-E) or a NightOWL camera (Berthold LB 983 NC100) with an excitation wavelength of 488 and 450 nm, respectively. Fluorescence emission was detected at 550 nm. Z-stack images were generated through confocal microscopy to remove the out of focus signal collected within each individual image. In some experiments, bright field images were taken using transmitted light detection (TD) to show leaf and root structures.

### Determination of growth parameters

Plants were harvested at 14, 21 and 31 days post inoculation (dpi). Entire plants were weighed after drying in a 70 °C oven for 3 days. Physiological variation in growth parameters were determined by total fresh and dry weight and by plant height.

### Statistical analysis

qRT-PCR data are expressed as the mean of absolute quantification (copy number) ± standard deviation (SD) of 3 biological replicates. Comparisons of mean RNA abundance of BSMV:*iLOV* were conducted using one-way analysis of variance (ANOVA). Differences among means were analyzed using Tukey’s post hoc test at *p* values <.05. The significance test between treatments for physiological variation in growth parameters was determined by a Student’s *t* test. Significance was set at *p* values <0.05. Statistical analysis was performed using InStat 3.0. Graphs were made using GraphPad Prism 7.0.

## Results and discussion

The efficiency of BSMV inoculation to silence gene expression in cereals has been documented and demonstrated in several studies [2, 6, 10, 12, 13]. Despite its substantial advantages in time, expense and efficiency, the system still has several limitations including an uneven distribution of gene silencing as shown using phytoene desaturase as reporter gene [2, 14]. To explore the potential use of BSMV for gene overexpression in different wheat tissues, a small gene (330 bp) encoding iLOV was cloned into a BSMV vector to inoculate *N. benthamiana* leaves via agro-infiltration.

**Fig. 2:**
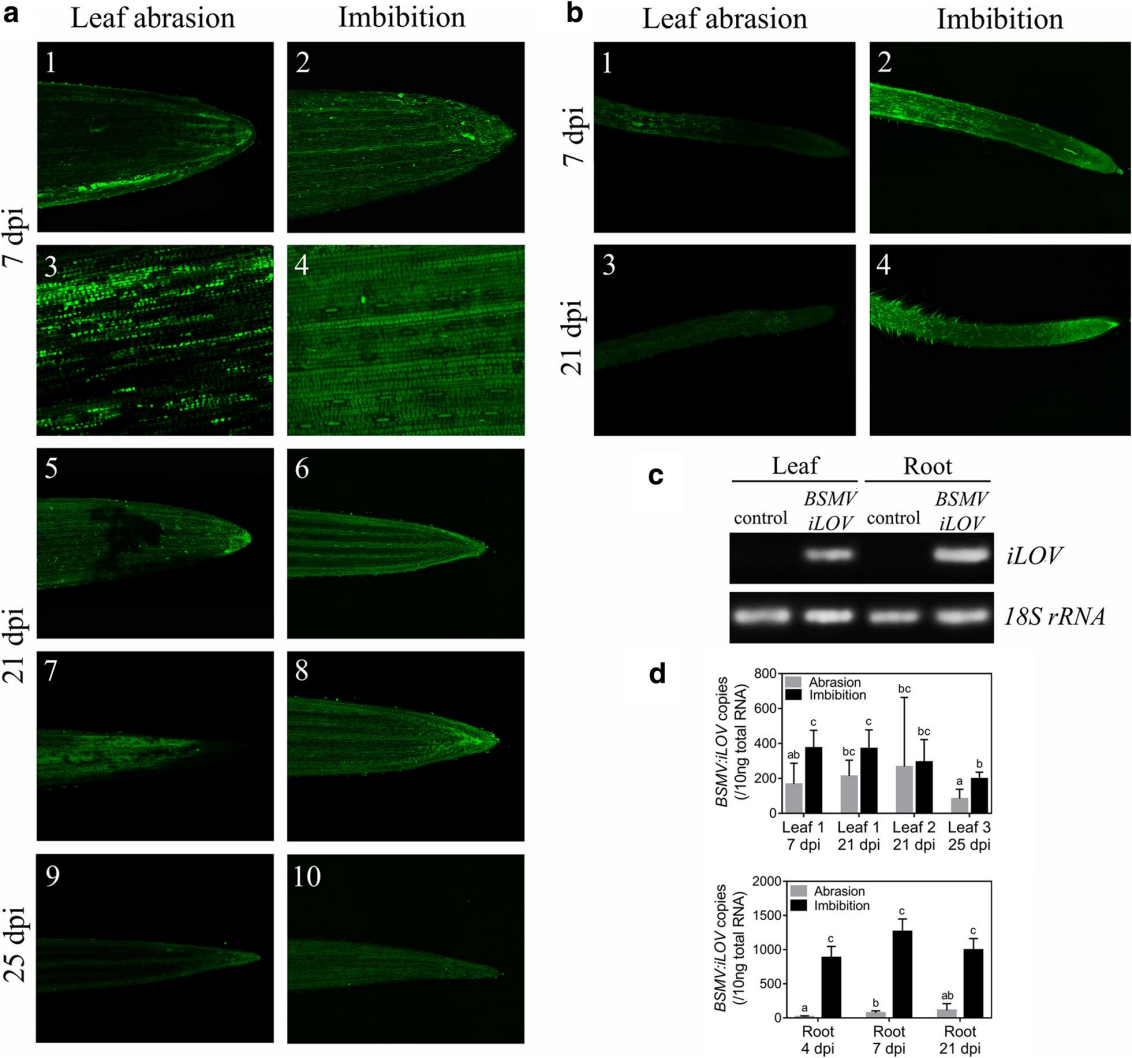
Expression of iLOV in wheat roots and leaves. **a** iLOV fluorescence images of leaves of BSMV:*iLOV* infected plants at 7 dpi (*1*–*4*), 21 dpi (*5*–*8*) and 25 dpi (*9*–*10*). A close-up is shown to see individual cells from the first leaves at 7 dpi using the scratching method (*3*) or the imbibition method (*4*). **b** iLOV fluorescence images of roots of BSMV:*iLOV* infected plants at 7 dpi (*1*–*2*) and 21 dpi (*3*–*4*). **c** Presence of *iLOV* region within the BSMV:*iLOV* RNA in leaves and roots at 7 dpi detected by RT-PCR. **d** Quantification of the *iLOV* region in leaves and roots at various days post infection by qRT-PCR. The copy number is calculated for the amount of RNA used in the qRT-PCR reaction using a calibration curve with known amounts of *iLOV* cDNA. Values represent mean ± SD (N  =  3). Different letters indicate significant differences between groups (p < 0.05)

After infiltration with an equal amount of *A. tumefaciens* harboring the three BSMV cDNA α, β and γ:*iLOV*, infected *N. benthamiana* plants started to develop yellow spots on infiltrated leaves and mild mottling on the upper uninfiltrated *N. benthamiana* leaves at 7 days post inoculation (dpi) (Additional file 2: Fig. S2). Since this phenotype indicates the presence of viral particles and systemic movement of BSMV from the infiltrated leaves, iLOV fluorescence in *N. benthamiana* leaves was visualized using a fluorescence camera (Berthold NightOWL) to confirm the infection with BSMV and iLOV expression (Additional file 2: Fig. S2). Infected leaves were ground to extract the BSMV particles. As mechanical transmission of BSMV via leaf abrasion is the recommended inoculation method to infect wheat plants, 7-day old wheat leaves were mechanically inoculated with *the N. benthamiana* leaf homogenate containing BSMV:*iLOV*. The presence of BSMV infection in inoculated wheat plants was observed from iLOV fluorescence using confocal microscopy 7 days after mechanical inoculation. Our result shows that iLOV is weakly expressed in both leaves and roots infected by mechanical inoculation but the signal distribution is very uneven with many cells showing no fluorescence signal (Fig. 2a panels 1 and 3, b panel 1). These results confirm the uneven distribution of BSMV particles throughout tissues as shown previously in different studies [1, 6, 10, 13, 15, 16]. Similar results were also observed in studies exploiting the potential of wheat streak mosaic virus-based vectors to overexpress GFP or beta-glucuronidase (GUS) using leaf abrasion [5, 17]. Ma et al. [18] reported that the effectiveness of BSMV expression in wheat was strongly related to the location of inoculation and the development stage of the plant. During seed imbibition, we expect that viral infection will be possible as soon as roots and leaves emerge. Although the mechanism of initial viral infection is still poorly understood [19], previous studies described that contact transmission is an important factor in the epidemiology of BSMV in the field [1, 20]. At the molecular level, the triple gene block proteins (TGB1, TGB2 and TGB3) present in most plant viruses including BSMV (encoded by the β genome) have been reported to play multiple functions in viral infection and propagation in the plant [1, 21, 22]. The interaction of the TGB1 protein with the TGB2 and TGB3 proteins is an important step to bind cell walls and cytoplasmic membranes and facilitate the movement of the viral nucleoprotein complex through the plasmodesmata to adjacent cells [22]. Furthermore, full systemic infection of plants by plant viruses can occur through roots by pouring infected sap on the soil or culture solutions in which they were growing indicating that simple contact may be sufficient for infection [20, 23]. We thus tested the possibility of incubating wheat seeds at the imbibition stage. Wheat seeds were germinated in distilled water with the *N. benthamiana* homogenate containing the viral particles carrying BSMV:*iLOV*. At 7 dpi, the distribution of iLOV fluorescence was observed throughout the leaf and root tissues and most if not all individual cells expressed iLOV (Fig. 2a panels 2 and 4, b panel 2). There is still some variation in signal intensity which may reflect the tissue structure and slight variation in the cell’s ability to replicate the viral RNAs or translate the different genes. However, most cells appear to translate the reporter gene efficiently. The progression of viral infection in the inoculated first leaf is presented in panels 5 and 6 after 21 dpi. The fluorescence signal has progressed throughout the leaf using the abrasion method but there is still an uneven signal with patches of stronger signal (Fig. 2a panel 1 vs 5). The iLOV signal for the imbibition method is similar to the one seen at 7 dpi (Fig. 2a panel 2 vs 6). The signal progression in the second leaf (21 dpi) and the emerging third leaf (25 dpi) is very weak throughout the leaf using the abrasion method (Fig. 2a panels 7 and 9) while the signal observed in the second and third leaf using the imbibition method is similar to the one observed in the first leaf (Fig. 2a panels 6, 8 and 10). BSMV:*iLOV* propagation in roots was also observed at 21 dpi. Unlike the roots infected via leaf abrasion (Fig. 2b panel 3), strong iLOV fluorescence is evenly emitted in roots infected via seed imbibition (Fig. 2b panel 4). These results demonstrate that the propagation of BSMV to different tissues through neighboring cells is relatively slow, suggesting that inoculation via leaf abrasion could require at least one generation to exhibit the phenotype of interest at whole plants level [1, 6], while the BSMV propagation via the imbibition method progressed at a faster rate possibly by exploiting the vascular tissues more efficiently.

To compare the level of expression in the different tissues, the *iLOV* RNA region was analyzed by RT-PCR to confirm the specificity of *iLOV* amplification products (Fig. 2c) and quantified by qRT-PCR (Fig. 2d). While inoculation of BSMV:*iLOV* into wheat plants either via seed imbibition or leaf abrasion allows the overexpression of iLOV (Fig. 2d), quantification analysis revealed a higher abundance of BSMV*:iLOV* copies in the first leaf of plants infected via seed imbibition at 7 dpi (Fig. 2d, upper panel). The level of BSMV*:iLOV* RNA at 21 days in leaf 1 is similar to the one at 7 days. In leaf 2, the level of BSMV*:iLOV* RNA is similar at 21 dpi between the two methods. However, the large standard error in the leaf treated with the abrasion method reflects a large variation in RNA copy number suggesting that viral propagation is uneven between the three different leaves used for analysis. This result is in agreement with the confocal analysis (Fig. 2a). The BSMV*:iLOV* abundance in the emerging third leaf was also determined by qRT-PCR. Although the quantification of BSMV:*iLOV* copies in the third leaf shows less variability, the seed imbibition method allows for a higher abundance of BSMV:*iLOV* in the growing leaf at 25 dpi suggesting that viral propagation is more efficient with the imbibition method. Root tissues were also harvested to verify the infection and spread of BSMV:*iLOV* in plants infected either via leaf abrasion or seed imbibition. Analysis by qRT-PCR reveals that BSMV:*iLOV* RNA is significantly more abundant in roots infected via seed imbibition, in contrast to leaf abrasion as soon as 4 dpi. This suggests that the imbibition method could be particularly useful to study the physiological impact of gene overexpression or RNAi during the early stages of plant growth and as early as 4 dpi.

**Fig. 3:**
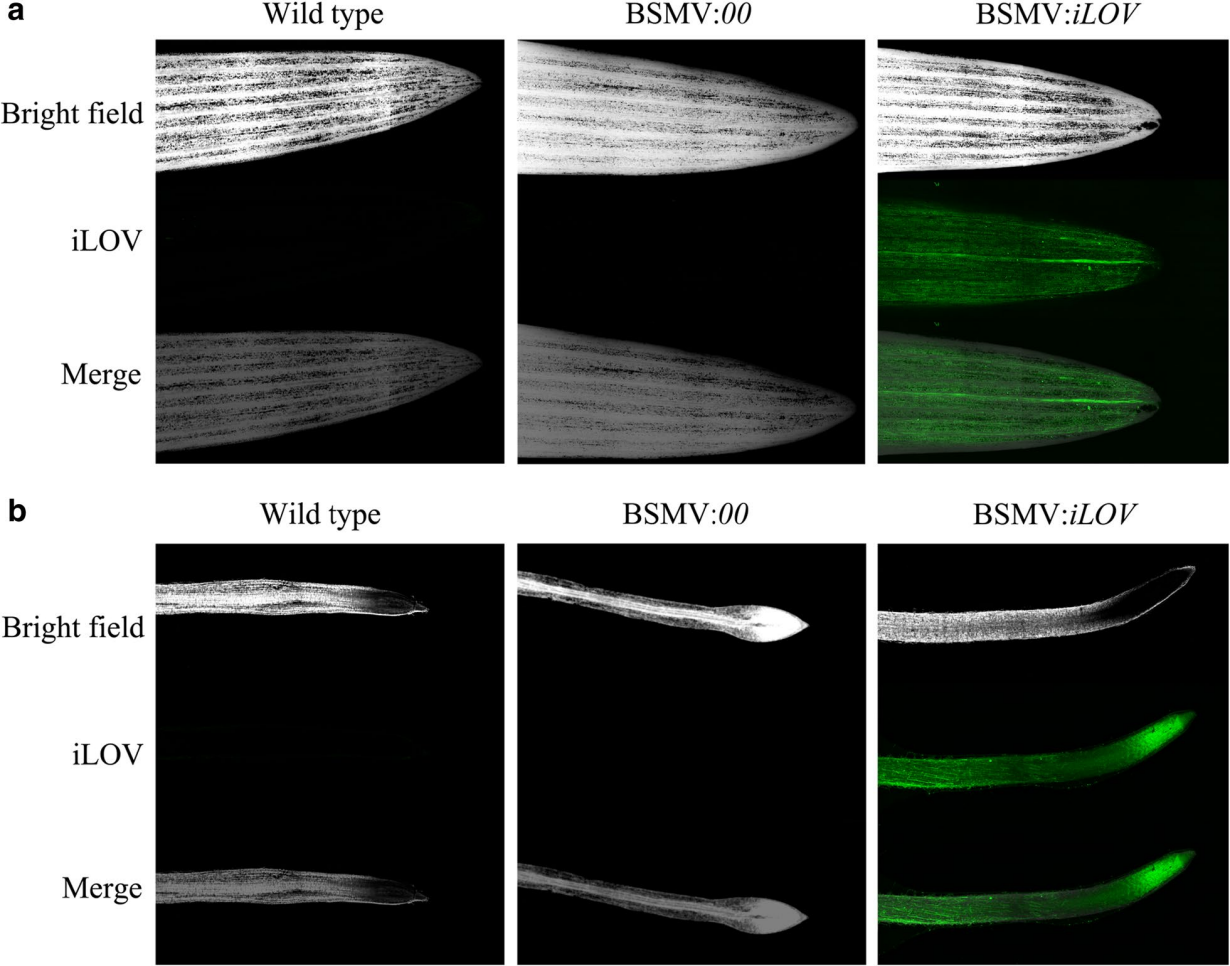
Expression of iLOV in leaves and roots of wheat plants. **a** leaves, **b** roots. Brightfield images (*top*); iLOV fluorescence images (*middle*) and merged images (*bottom*) are shown. Wheat seeds were imbibed for 3 days in extract of wild type-uninfected tobacco (Wild-type, *left*), tobacco infected with empty BSMV vector (BSMV:*00*, *middle*) or with BSMV:*iLOV* (*right*)

To confirm that the imbibition method does not cause a background fluorescence signal, two different controls were made (Fig. 3). Plants were either incubated with uninfected or BSMV wild type-infected (BSMV:*00*) *N. benthamiana* leaf homogenates. In both cases, no fluorescence signal was observed in the leaf (Fig. 3a, left and middle panels respectively) or root tissues (Fig. 3b, left and middle panels respectively), while a strong fluorescence signal was detected in both leaf and root tissues infected with BSMV:*iLOV* (right panels in Fig. 3a, b, respectively).

In previous studies performing virus-induced gene silencing (VIGS) with the scratching method, the peak effect of gene silencing was observed at 20 dpi indicating that the imbibition method allows gene expression and function studies at a much earlier growth development stage. Together, these results confirm the presence of iLOV in root and leaf tissues, and suggest that the seed imbibition method greatly improves the distribution of BSMV and expression of iLOV.

**Fig. 4:**
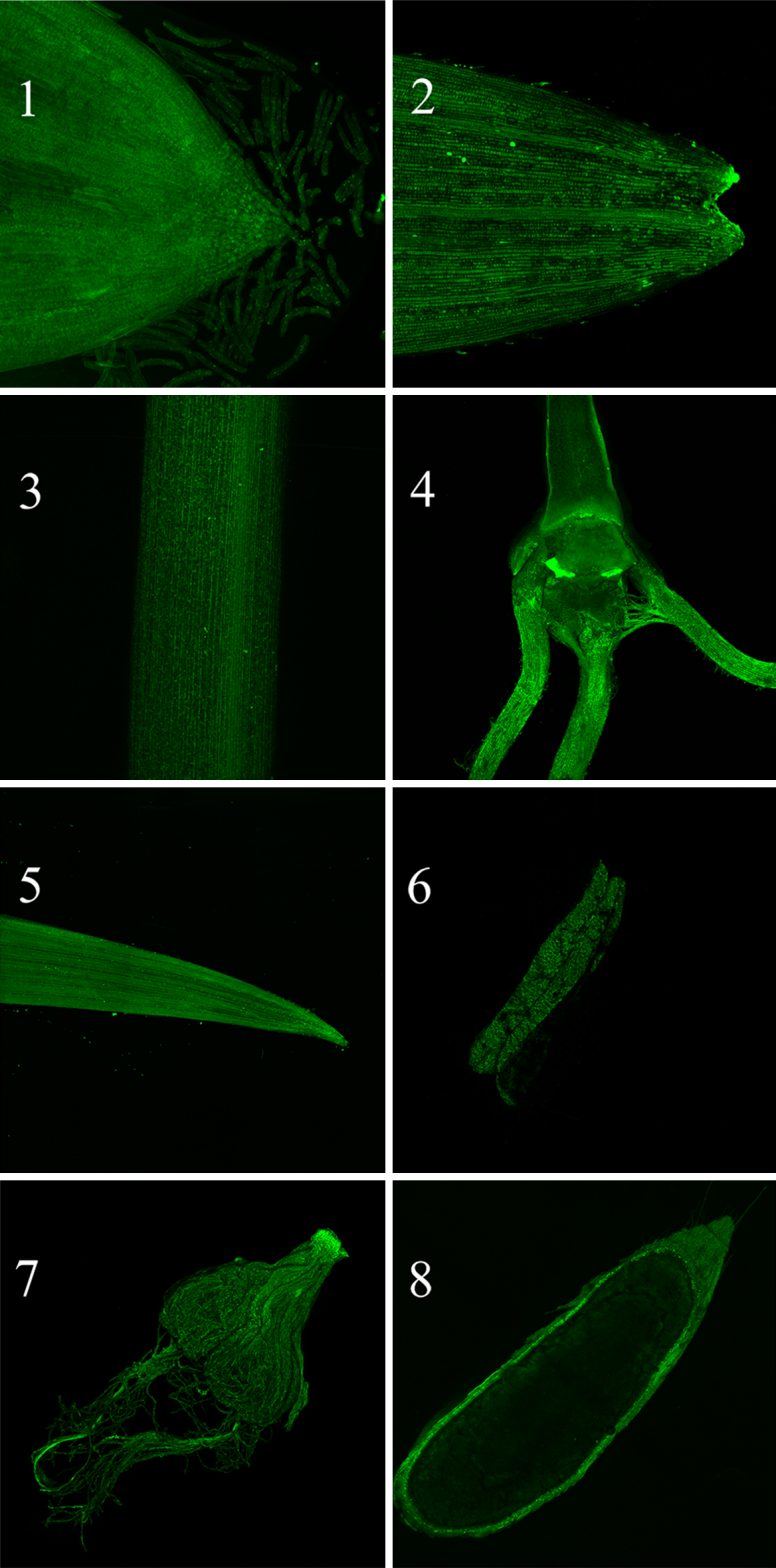
Expression of iLOV in different tissues/organs of BSMV:*iLOV* infected plants. Root tip (*1*); leaf tip (*2*); shoot (*3*); crown (*4*); flag leaf (*5*); anther (*6*); immature seed (*7*); mature seed (*8*)

It has been reported that BSMV could mediate gene expression in different wheat organs [24]. However, a visually discernible signal throughout different organs/tissues is needed to support the use of BSMV in studies examining the effect of genes expressed in these organs. The progression of virus infection was measured over several weeks in different wheat tissues to verify the stability of the system and the expression in other tissues up to the flowering and seed maturation stages. Figure 4 shows a uniform fluorescence of iLOV in different organs or tissues (root tip, leaf tip, crown, flag leaf, anthers, immature seed and mature seed) of wheat infected via seed imbibition. Although a small level of auto fluorescence was observed on the coat of wild type mature seed (not shown), BSMV:*iLOV* infected mature seeds emitted a strong fluorescence on the coat and in the endosperm (Fig. 4, panel 8), indicating the presence of iLOV expression in mature seeds. This is in agreement with previous studies showing that gene silencing can be propagated to the next generation [1, 6].

**Fig. 5:**
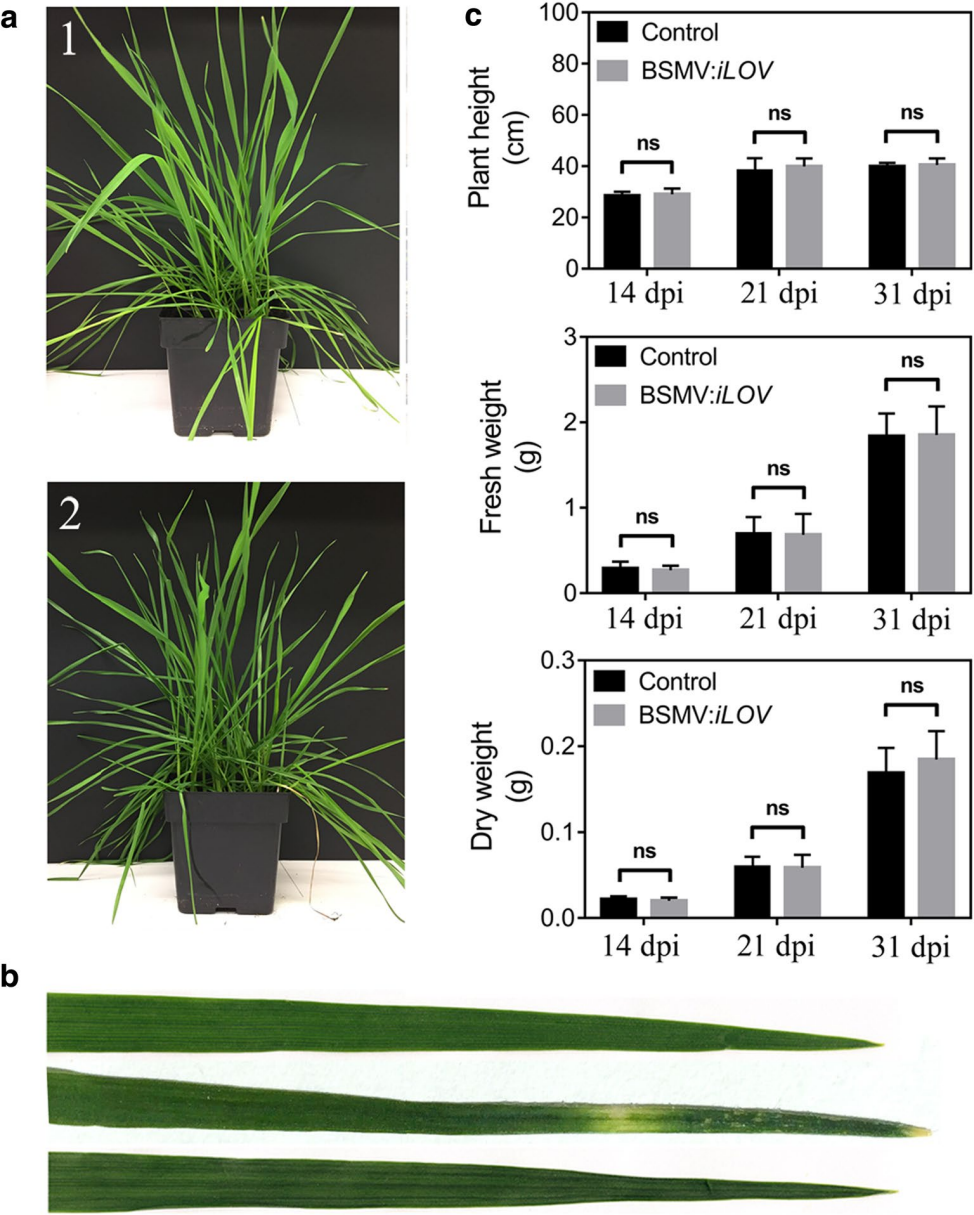
Effect of BSMV on wheat plant growth. **a** Phenotypes of uninfected (*1*) and BSMV:*iLOV* infected (*2*) wheat plants at 31 dpi. **b** Phenotype of uninfected (*up*) and BSMV:*iLOV* infected wheat leaves via leaf abrasion (*middle*) or seed imbibition (*down*) methods at 31 dpi. **c** Physiological variation in growth parameters. Height, fresh weight and dry weight of whole plants (n = 30) were measured at 14, 21 and 31 dpi. Control plants were inoculated with leaf homogenates of uninfected wild-type *N. benthamiana*. BSMV:*iLOV* plants were inoculated with leaf homogenates of BSMV:*iLOV* infected *N. benthamiana*. *ns* non significant difference

Previous reports indicated that virus inoculation could alter photosynthesis in plants leading to a reduction of chlorophyll content and growth rate [14, 25, 26]. By inoculating barley plants via leaf abrasion, Liang et al. [14] stated that inoculation of BSMV reduced significantly the growth rate of barley plants and accelerated leaf senescence. Since the imbibition method did not cause any apparent symptoms (Fig. 5a, b) comparatively to the leaf abrasion method where necrotic lesions were apparent on infected tissues in wheat or tobacco (Fig. 5b; Additional file 2: Fig. S2), we evaluated the effects of BSMV inoculation by the imbibition method on the subsequent vegetative growth of plants at different growth periods (14, 21 and 31 dpi). The results show that inoculation of wheat plants by seed imbibition did not significantly affect the plant height and biomass as determined by fresh and dry weights (Fig. 5c). This suggests that seed imbibition could be a more appropriate method to investigate phenotypes of various genes related to development or stress responses. To illustrate the benefit of the imbibition method in dicot species, the expression of iLOV throughout leaf tissues of *N. benthamiana* and *A. thaliana* is illustrated in Additional file 3: Fig. S3.

**Fig. 6:**
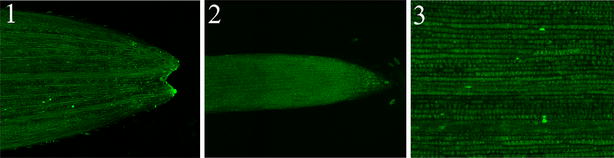
Expression of iLOV in leaf (*panel 1*) and root (*panel 2*) of the next generation of BSMV-infected wheat plants at the seedling stage (7 days). Close-up to see individual cells in the leaf tissue (*panel 3*)

Since the presence of iLOV was detected in mature seeds (Fig. 4, panel 8), the expression of iLOV in the next generation was visualized at the seedling stage to confirm viral transmission. Confocal microscopy revealed the fluorescence of iLOV both in leaves and root tissues of infected plants (Fig. 6, panels 1 and 2). A close-up of iLOV fluorescence shows that BSMV:*iLOV* is uniformly spread throughout the leaf tissue, which is relatively similar to the results of inoculation obtained via imbibition method in the first plant generation (Fig. 2).

**Fig. 7:**
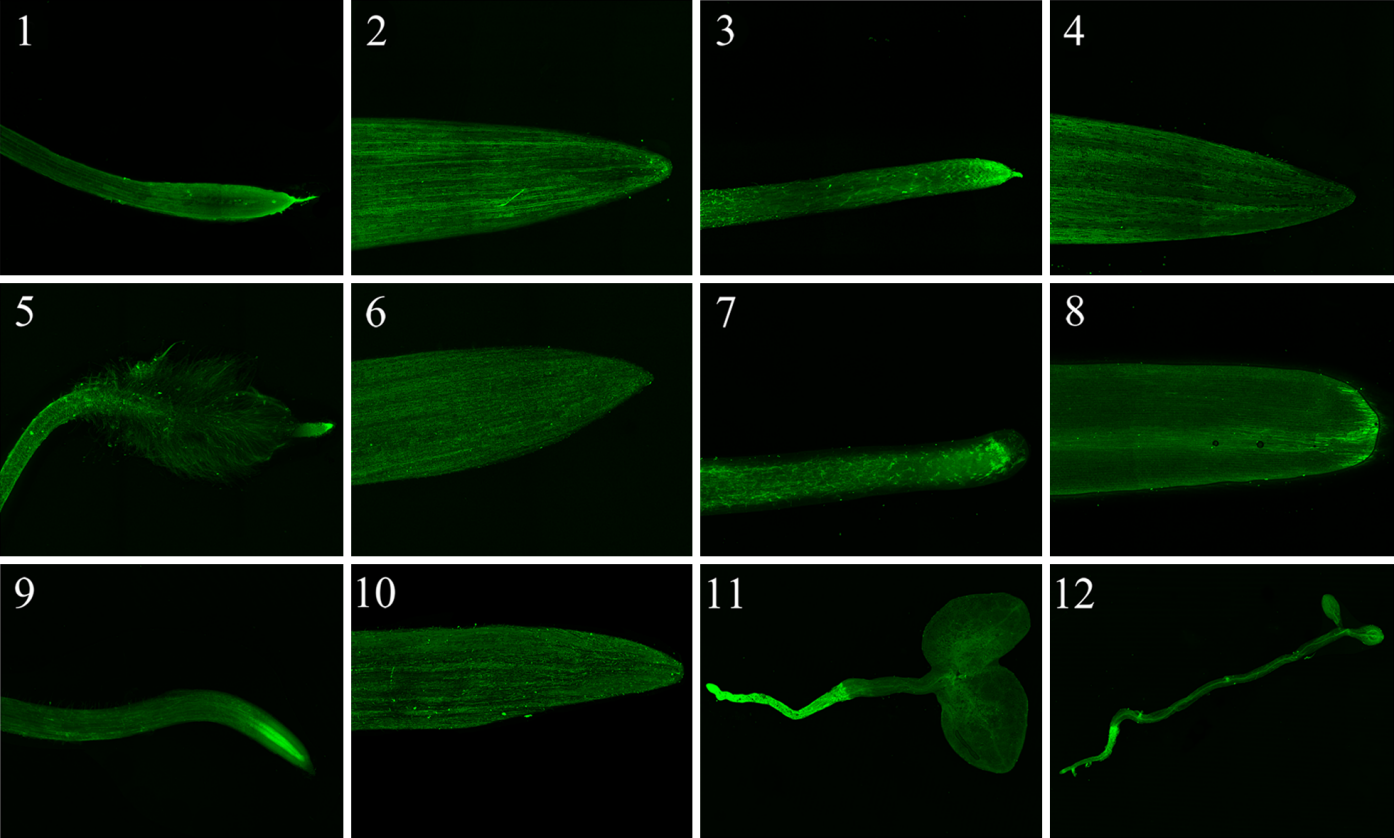
Expression of iLOV in leaves and roots of different wheat cultivars, monocot and dicot species at 7 dpi. Wheat cv. Atlas 66 root (*1*), leaf (*2*); wheat cv. Norstar root (*3*), leaf (*4*); *B. distachyon* root (*5*), leaf (*6*); *Z. mays* root (*7*), leaf (*8*); *H. vulgare* L. root (*9*), leaf (*10*); *N. benthamiana* (*11*); *A. thaliana* (*12*)

Previous studies have reported that the ability of BSMV to infect host plant depends on genotypes [1, 6]. The potential of BSMV to infect different wheat genotypes via seed imbibition was examined. Different wheat cultivars (Atlas 66, Norstar) were inoculated via seed imbibition with *N. benthamiana* containing particles of BSMV:*iLOV.* Visualization of infected wheat plants by confocal microscopy reveals that iLOV was uniformly expressed in roots and leaves of both wheat cultivars (Fig. 7, panels 1–4). This suggests that inoculation of BSMV by seed imbibition can be used with different wheat cultivars. Mechanical transmission of BSMV to over 250 plant species including both monocot and dicots species has been reported [1, 27]. We thus investigated the ability of BSMV to infect different monocot and dicot species via seed imbibition. Seeds of *B. distachyon*, maize and barley were germinated on Petri dishes with water containing the *N. benthamiana* homogenate. Roots and leaves were imaged by confocal fluorescence microscopy at 7 dpi. The green fluorescence of iLOV was distributed uniformly in roots and leaves of all infected monocot plant species (Fig. 7, panels 1–10). By inoculating seeds of dicot species (*N. benthamiana* and *A. thalian*a) with *the N. benthamiana* homogenate containing BSMV:*iLOV* viral particles, we can observe that iLOV is expressed throughout the whole plant with a stronger signal in roots compared to leaves (Fig. 7, panels 11 and 12). However, the signal is uniform within a same tissue and in the different leaves either at the two leaf stage (Fig. 7, panels 11 and 12) or when additional leaves emerge (Additional file 3: Fig. S3). These results show that inoculation via seed imbibition allows uniform distribution of BSMV in different monocot and dicot species allowing gene function experiments to be performed with confidence.

## Conclusion

In this study, we have developed a low-cost high-throughput inoculation method allowing rapid and uniform gene expression in different plants based on the barley stripe mosaic virus. Using this new approach, we demonstrated that inoculation greatly improves transfection compared to the traditional leaf abrasion method, and allows efficient and stable viral propagation in different tissues during the first generation as evidence with the iLOV fluorescent reporter. Hence, this protocol could help researchers to take full advantage of the BSMV system which is a powerful functional genomics tool for gene function characterization using gene overexpression or gene silencing in different plant species, particularly during different stages of plant growth.

## Additional files

**Additional file 1.** Schematic representation of BSMV:*iLOV* construct. Detail of LIC cloning is illustrated.

**Additional file 2.** (1) Uninfected *N. benthamiana* plant. (2) Agrobacterium mediated BSMV:*iLOV* infection in *N. benthamiana* plant. *N. benthamiana* leaves were inoculated by agro-infiltration with an equal amount of *Agrobaterium* mixtures harboring the pCaBS-α, pCaBS-β and pCaBS-γb:*iLOV*. (3) iLOV fluorescence imaged by a NightOWL camera in an uninfected leaf. (4) iLOV fluorescence imaged by a NightOWL camera in a BSMV:*iLOV* infected leaf.

**Additional file 3.** Photograph of BSMV-infected tobacco via seed imbibition. (2) Photograph of BSMV-infected *A. thaliana* via seed imbibition. (3) iLOV fluorescence imaged by a confocal microscopy in a BSMV:*iLOV* infected tobacco via seed imbibition. (4) iLOV fluorescence imaged by a confocal microscopy in a BSMV:*iLOV*-infected *A. thaliana* via seed imbibition.
