# Supplementary figures and images for: A rapid and efficient method for uniform gene expression using the barley stripe mosaic virus
Source: Plant Methods. 2017 Apr 11;13:24. doi: 10.1186/s13007-017-0175-5 (PMC5387290; doi:10.1186/s13007-017-0175-5)

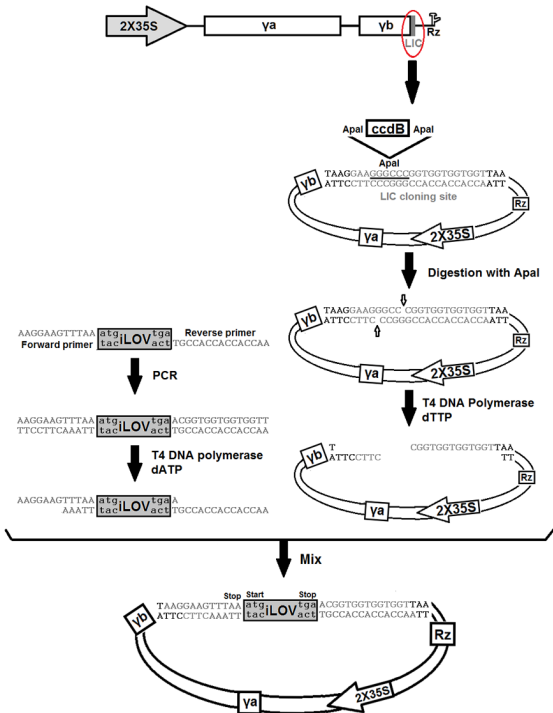

Figure S1

Supplement: Supplementary file 1 — Additional file 1. Schematic representation of BSMV:iLOV construct. Detail of LIC cloning is illustrated. [file 13007_2017_175_MOESM1_ESM.pdf]

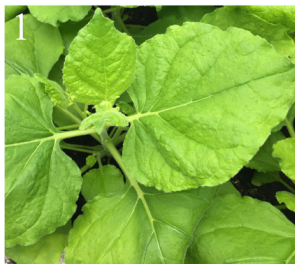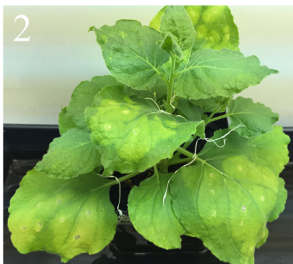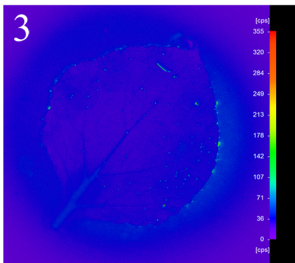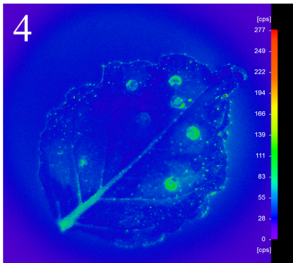

Figure S2

Supplement: Supplementary file 2 — Additional file 2. (1) Uninfected N. benthamiana plant. (2) Agrobacterium mediated BSMV:iLOV infection in N. benthamiana plant. N. benthamiana leaves were inoculated by agro-infiltration with an equal amount of Agrobaterium mixtures harboring the pCaBS-α, pCaBS-β and pCaBS-γb:iLOV. (3) iLOV fluorescence imaged by a NightOWL camera in an uninfected leaf. (4) iLOV fluorescence imaged by a NightOWL camera in a BSMV:iLOV infected leaf. [file 13007_2017_175_MOESM2_ESM.pdf]

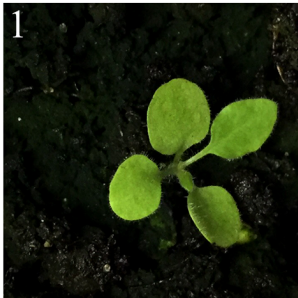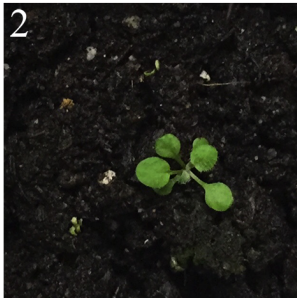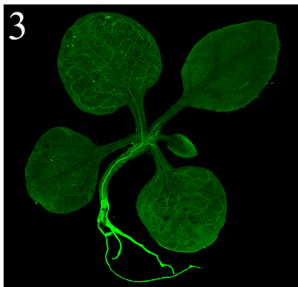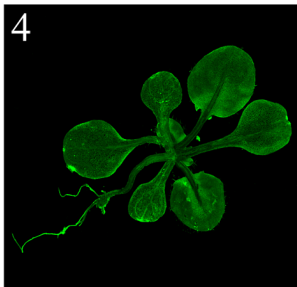

Figure S3

Supplement: Supplementary file 3 — Additional file 3. Photograph of BSMV-infected tobacco via seed imbibition. (2) Photograph of BSMV-infected A. thaliana via seed imbibition. (3) iLOV fluorescence imaged by a confocal microscopy in a BSMV:iLOV infected tobacco via seed imbibition. (4) iLOV fluorescence imaged by a confocal microscopy in a BSMV:iLOV-infected A. thaliana via seed imbibition. [file 13007_2017_175_MOESM3_ESM.pdf]
